# Hydrostatic pressure of the renal pelvis as a radiation-free alternative to fluoroscopic nephrostogram following percutaneous nephrolithotomy

**DOI:** 10.1186/s12894-023-01225-6

**Published:** 2023-03-28

**Authors:** Nici Markus Dreger, Dominik Stapelmann, Patrick Rebacz, Stephan Roth, Alexander Sascha Brandt, Friedrich-Carl von Rundstedt, Stephan Degener

**Affiliations:** 1 Department of Urology, Helios University Hospital Wuppertal, Witten/Herdecke University, Heusnerstrasse 40, 42283 Wuppertal, Germany; 2 Department of Radiology, St. Franziskus-Hospital Münster, 48145 Münster, Germany; 3 Didactics and Educational Research in Healthcare, Department of Medicine, Faculty of Health, Witten/Herdecke University, 58455 Witten, Germany; 4 Urologisches Zentrum Euregio, Humboldtstrasse 1, 52152 Simmerath, Germany

**Keywords:** Renal pelvis pressure, Radiation-free, Nephrostogram, Percutaneous nephrolithotomy

## Abstract

**Background:**

We evaluated the hydrostatic pressure of the renal pelvis (RPP) as a radiation-free alternative to fluoroscopic nephrostogram to assess ureteral patency after percutaneous nephrolithotomy (PCNL).

**Methods:**

Retrospective non-inferiority study analyzing 248 PCNL-patients (86 female (35%) and 162 males (65%)) between 2007 and 2015. Postoperatively, RPP was measured using a central venous pressure manometer in cmH_2_O. The primary endpoint was to assess RPP depending on the patency of the ureter and the nephrostomy tube removal. Secondary, the upper limit of normal RPP of $\le$ 20 cmH_2_O was assessed as an indicator of an unobstructed patency.

**Results:**

The median procedure duration was 141 min (112–171.5) with a stone free rate of 82% (n = 202). RPP was significantly higher in patients with obstructive nephrostogram with 25.0 mmH_2_O (21.0–32.0) versus 20.0 mmH_2_O (16.0–24.0; p < 0.001). The pressure was lower in successful nephrostomy removal with 18 cmH_2_O (15–21) versus 23 cmH_2_O (20–29) in the leakage group (p < 0.001). The analysis of a cut-off of $\le$ 20 cmH_2_O showed a sensitivity of 76.9% (95% CI [60.7%; 88.9%]) and a specificity of 61.5% (95% CI [54.6%; 68.2%]). The negative predictive value was 93.4% (95% CI: [87.9%; 97.0%]) and the positive predictive value 27.3% (95% CI [19.2%; 36.6%]). The accuracy of the model showed an AUC = 0.795 (95% CI [0.668; 0.862]).

**Conclusion:**

The hydrostatic RPP seems to allow a bedside evaluation of ureteral patency after PCNL.

## Introduction

Percutaneous nephrolithotomy (PCNL) has become the gold standard of treatment for large renal stones with stone-free rates (SFR) up to 90% [1–3]. Although there is a world-wide and strong trend towards tubeless PCNL, [4] the postoperative nephrostomy tube (NT) placement in the renal pelvis remains the standard of care in many institutions.

Despite high success, PCNL is associated with minor complications such as urinary tract infection and urinoma as well as serious complications such as adjacent organ injury, significant blood loss or urosepsis [5]. One common minor complication is prolonged urinary leakage from the nephrostomy site after the removal of the NT [5, 6]. The predominant imaging reference standard to assess ureteral patency is fluoroscopic nephrostogram (FN) [6]. It is usually performed at postoperative day (POD) 1–2 with iodinated contrast medium through the indwelling NT. This enables decisions on whether the NT can be removed to be made in real time; however, it requires the use of ionizing radiation (IR) and must be performed in a dedicated fluoroscopy workplace.

Because of the growing evidence on malignant and non-malignant effects of IR, the aim of any procedure should be to reduce patient radiation exposure to as low as reasonably achievable [7]. In PCNL patients, it is of particular interest to reduce the radiation dose received because of their unavoidable exposure during diagnosis and treatment. [8].

Pressure-controlled nephrostogram (PCNG) was introduced by Robert H. Whitaker to assess the dynamics of the upper urinary tract after renal operations such as ureteral or ureteropelvic stenosis [9, 10] methods that work without IR, with less equipment and therefore also at a lower cost. Normal renal pelvic pressure (RPP) was shown to range between 12 and 20 cmH_2_O, whereas values above 20 cmH_2_O indicate a ureteral obstruction [11, 12].

The aim of this study was to prove the benefit of PCNG for the first time in PCNL, to evaluate the historically proven RPP threshold of 20 cmH_2_O for a safe removal of the NT, [12] and to investigate whether a postoperative RPP-measurement is as effective as FN after PCNL.

## Material and methods

This single site retrospective study included all patients who underwent PCNL in Helios University Hospital Wuppertal urology department between 2007 and 2015. This study was conducted with approval from the Institutional Review Board, and reported according to STROBE guidelines.

PCNL was done according to a standardized protocol, followed by FN and PCNG in all patients. Initially, a 7 French (F) ureteral catheter was placed cystoscopically to perform pyelography in each patient. Access to the renal pelvis was achieved in the prone position by ultrasound-guided puncture of the selected calix. A J-tip guidewire was placed through the needle and the fascia was cut by wire-guided Korth-knife. The nephrostomy tract was dilatated using an Amplatz-type dilator for single-step dilatation and an Amplatz sheath. All PCNL procedures were performed with 30 F nephroscope (Karl Storz, Tuttlingen, Germany). Stone-free status was confirmed both fluoroscopically and endoscopically and afterwards a balloon-tipped NT (20 F, 3 ml filling) was placed in the renal pelvis.

At POD 1 an abdominal X-ray was performed to exclude residual stone fragments and the intraoperatively placed ureteral catheter was removed.

Usually at POD 2 a FN was performed to confirm ureteral patency (without any stent in the ureter) and to decide whether the NT could be removed (in the case of ureteral obstruction, the NT was left in place). The RPP was measured afterwards independently of that decision. Prolonged urine leakage (PUL) was defined as a persistent leakage > 12 h, requiring repeated dressing changes due to saturation. A ureter stent in case of persistent leakage was routinely placed after three days or when patients showed clinical signs of obstruction (pyelonephritis and/or renal failure).

**Fig. 1:**
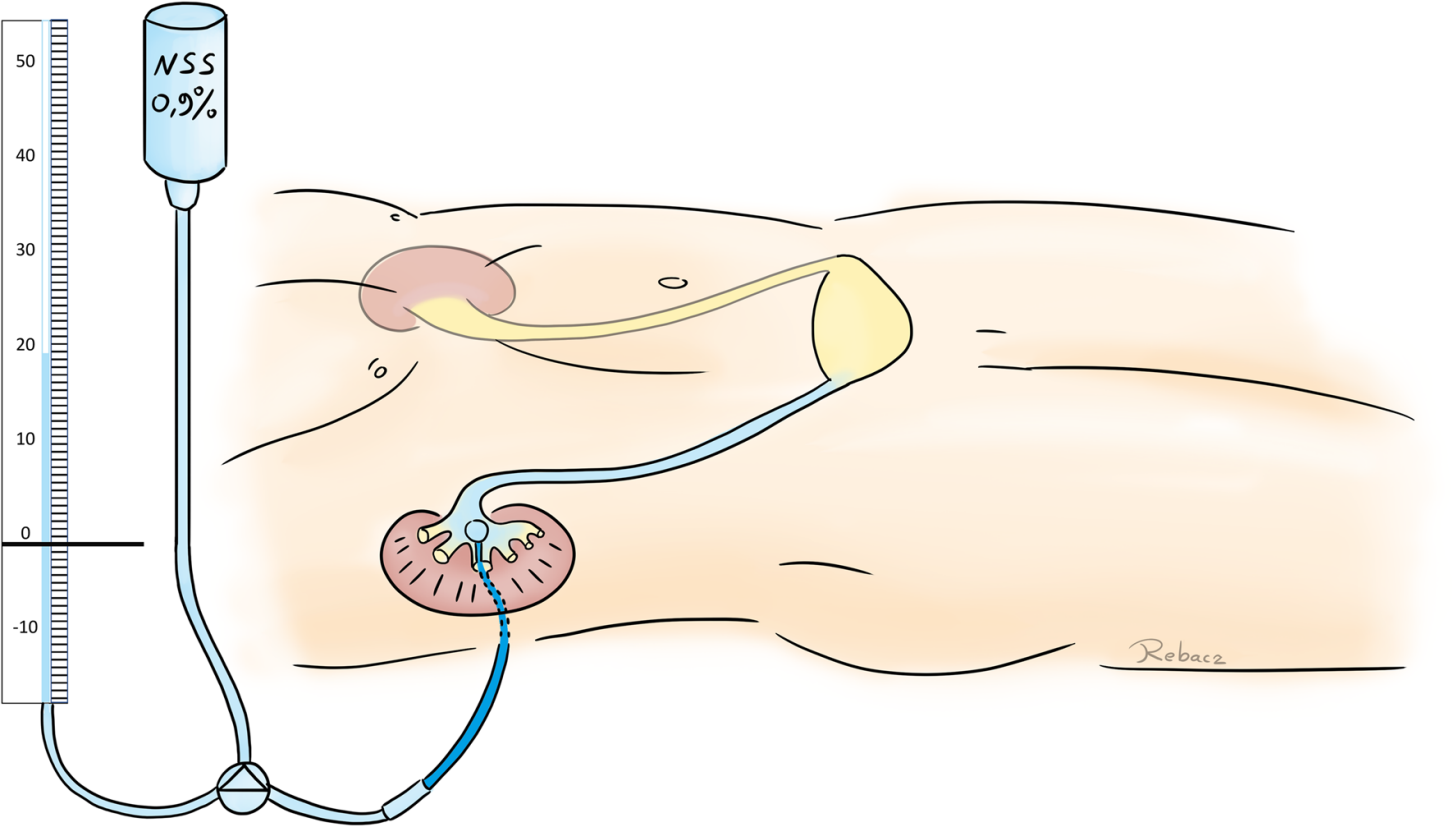
Method of a pressure-controlled nephrostogram using normal saline 0.9% as infusion fluid via indwelling nephrostomy, a normal manometer to measure the pressure in cmH_2_O and a regular three-way-tap. The zero line is localized at the level of the kidney

For RPP, a regular normal saline (NSS) drip (hanging 80 cm above the kidney level) was attached through a regular infusion tube (sterile i.v.-set, single use) to the NT and to a regular small diameter water column manometer (typically used for central venous pressure; CVP) using a three-way tap. The zero line of the manometer was placed at the level of the kidney. First, the manometer was filled with the NSS via gravity (three-way tap closed to NT). Then the tap was closed to the manometer and opened to the NT. The NT and renal pelvis were also filled via gravity. When the inflow slowed down due to a pressure equilibrium in the renal pelvis, the tap was closed to the tube of the drip, resulting in a connection of the fluid in the manometer and in the NT/renal pelvis. Consecutively, the fluid could be observed oscillating in the manometer where the height of the column indicates the RPP (see Fig. 1).

The primary endpoint of this study was to assess the RPP level (measured in cmH_2_O) in cases of disturbed and undisturbed patency of the ureter after PCNL and to analyze differences in RPP depending of (un)successful NT removal. Success was defined as closure of the skin without prolonged urine leakage.

The secondary endpoint was to evaluate the physiological upper limit of normal RPP of $\le$ 20 cmH_2_O ^10, 11^ as an indicator of an unobstructed patency of the ureter and to assess the diagnostic accuracy of the method. Consequently, we analyzed whether the RPP is a safe and reliable alternative to FN to decide whether the NT can be removed after PCNL.

Subgroups of participants who had PUL versus those with no leakage were derived at the endpoint of the study. Comparisons of continuous parameters were based on medians and quartiles (graphically charted on non-parametric box plots), whereas comparison of categorical parameters on absolute and appropriate relative frequencies. No adjustments were made for group imbalance. Wilcoxon as well as Fisher’s exact tests were applied to subgroup comparisons, with a p value of ≤ 0.05 indicating significant findings.

The sensitivity, specificity, positive predictive value (PPV) and negative predictive value (NPV) of the estimated threshold of RRP $\le$ 20 cmH_2_O were calculated. To assess the accuracy of the method, an analysis of the area under the receiver operating characteristic curve (AUC) was performed and classified according to the traditional academic point system.^12^ Pediatric patients and patients with missing RPP data were excluded from the analysis. We used SPSS version 23 (IBM Inc., Armonk, NY, U.S.A) for all statistical analyses.

## Results

Over the course of the observation period, we performed single-tract PCNL in 381 patients, of whom 248 (65%) were analyzed after excluding patients with missing data. Of the study population, 86 were female (35%) and 162 were male (65%), with a median age of 52 years (IQR 44–64). The median body-mass index (BMI) of the study population was 28.0 kg/m^2^ (24.6–32.1), and 54% (n = 135) of stones were located in the left kidney. Median stone size (defined as the largest diameter) was 19 mm (7–113). The predominant stone localization was the renal pelvis with 36.3% (n = 90). A total of 2.4% (n = 6) of stones were found in the upper calyx, 1.6% (n = 4) in the middle, and 10.5% (n = 26) in the lower calyx. Partial staghorn stones (renal pelvis + 1 calyx) were found in 11.3% (n = 28) and complete staghorn stones (renal pelvis +  > 1 calyx) in 7.7% (n = 19) of patients. Multiple kidney stones were diagnosed in 29.8% (n = 74). No patient had a medical history of ureterovesical reflux.

The median duration of the PCNL procedure was 141 min (112–171.5) with a SFR of 82% (n = 202) after the first procedure. In 10% of the patients (n = 24) a second PCNL was required to achieve stone-free status.

In all patients a fluoroscopic nephrostogram was performed to assess the ureteral patency, indicating that the NT could be removed. In 64% of patients (n = 159) the ureteral patency was demonstrated at the time of the first FN, in 89 patients (36%) repeat FN was required to exclude a persistent obstruction of the ureter.

The median radiation dose of one FN was 40.0 cGy*cm^2^ (19.3–81.0), resulting in a median cumulative radiation dose per patient of 50.0 cGy*cm^2^ (25.6–143.0). The NT remained in the kidney for a median of 4 days (3–6).

**Fig. 2:**
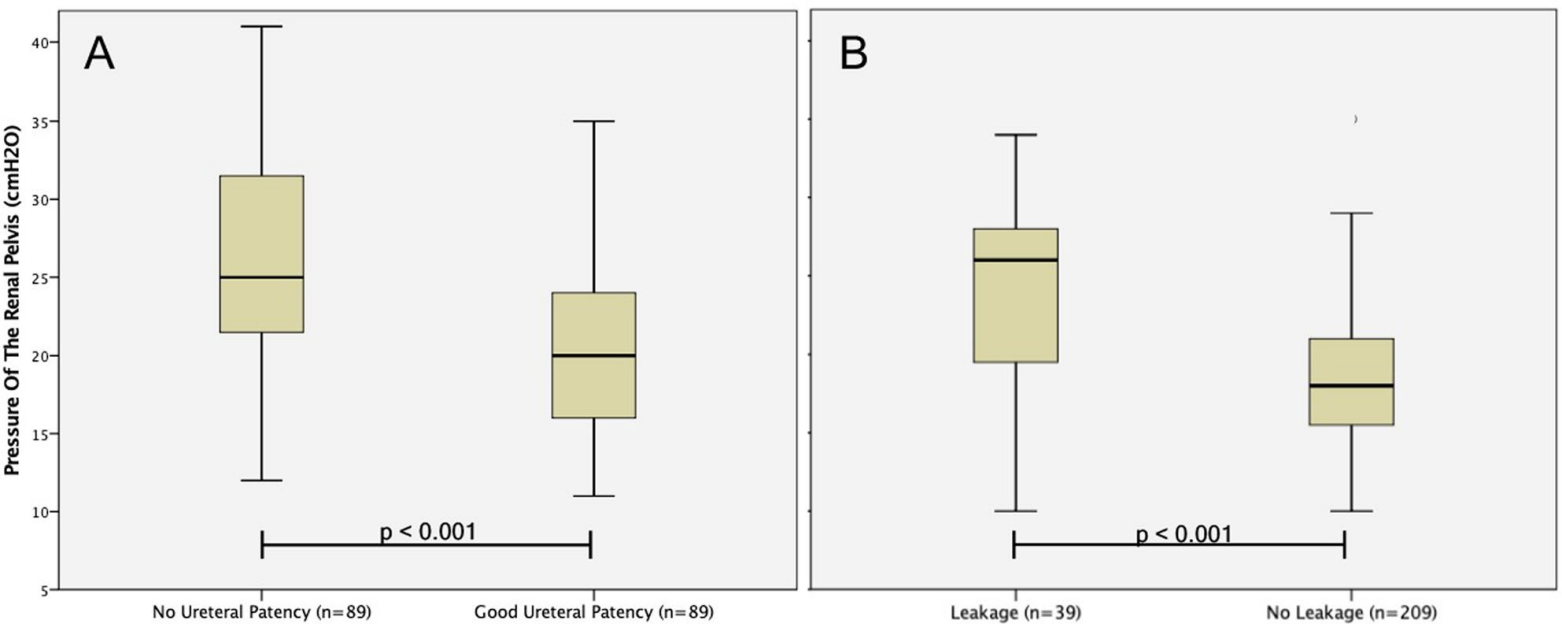
Box plot of **A** the RPP (cmH_2_O) in patients with and without adequate ureteral patency in post-PCNL fluoroscopic nephrostogram and **B** of the RPP after removal of the nephrostomy tube in patients with and without consecutive PUL; box 25/75th percentiles, line median, whiskers extend to the 1.5 interquartile range

With regards to the primary endpoint of this study, the median RPP was significantly lower in patients with an uncomplicated removal of the nephrostomy tube (n = 209; 84%) measuring18 mmH_2_O (15–21) vs. 23 mmH_2_O (20–29) in patients with prolonged urinary leakage (n = 39; 16%) from the access site (Fig. 2B). Table 1 gives a detailed comparison of the two groups.

**Table 1:** Demographic and procedure baseline parameters stratified for PUL. Absolute and relative frequencies for categorical variables, and medians and quartiles for continuous sociodemographic variables are provided

		Without PUL	PUL	p-Value
*Basic parameter*				
No. Patients	n = 248	n = 209 (84%)	n = 39 (16%)	p = 0.470^*^
Age	[y]	53 (43–65)	50 (46–58)	p = 0.518^#^
Sex	Male	n = 137 (55%)	n = 25 (10%)	p = 0.483^*^
	Female	n = 72 (29%)	n = 14 (6%)	
BMI	[kg/m^2^]	27.4 (24.3–31.2)	30.7 (27.8–36.0)	p < 0.001^#^
Max. Stone Size	[mm]	19 (14 – 26)	20 (14–33)	p = 0.443^#^
PCNL History	No	n = 165 (67%)	n = 32 (13%)	p = 0.753^*^
	Yes	n = 43 (17%)	n = 7 (3%)	
Procedure parameter				
Duration PCNL	[min]	141 (111–171)	138 (117–166)	p = 1.000^#^
Second look PCNL	n = 24	n = 21 (10%)	n = 3 (8%)	p = 0.454^*^
Stone free	n = 202	n = 174 (83%)	n = 28 (72%)	p = 0.067^*^
Time with NT	[d]	4 (3–5)	4 (3–7)	p = 0.064^#^
No. Nephrostograms	1	139	20	p = 0.111^*^
	2	52	13	
	3	14	6	
	4	4	0	
Cumulative radiation dose due to FN	cGy*cm^2^	47.3 (24.7–97.0)	134.6 (65.7–236.7)	p < 0.001^#^
Renal pelvis pressure	[mmH_2_O]	18 (15–21)	23 (20 – 29)	p < 0.001^#^
Post-PCNL ureter stenting	n = 7	–	n = 7 (18%)	–

^*^Exact Fisher tests

^#^two-sample Wilcoxon tests

Furthermore, we analyzed the difference in the RPP when FN had to be repeated, because the first one showed no ureteral patency (n = 89; 36%). In the case of an obstructive FN, the RPP was significantly higher with 25.0 mmH_2_O (21.0–32.0) compared to the secondary FN in those patients with 20.0 mmH_2_O (16.0–24.0) respectively (p < 0.001; Fig. 2A).

For the second endpoint we analyzed our hypothesized cut-off value of $\le$ 20 cmH_2_O. With the nephrostogram standard, 16% of patients (n = 39) in this study had urine leakage despite unobstructed passing of contrast in the nephrostogram. If the RPP with a cut-off value of 20 cmH_2_O would have been used instead of the FN, only 4% of patients (n = 9) would have had a urine leakage after removal of the NT corresponding in a higher accuracy when predicting a successful nephrostomy removal.

**Fig. 3:**
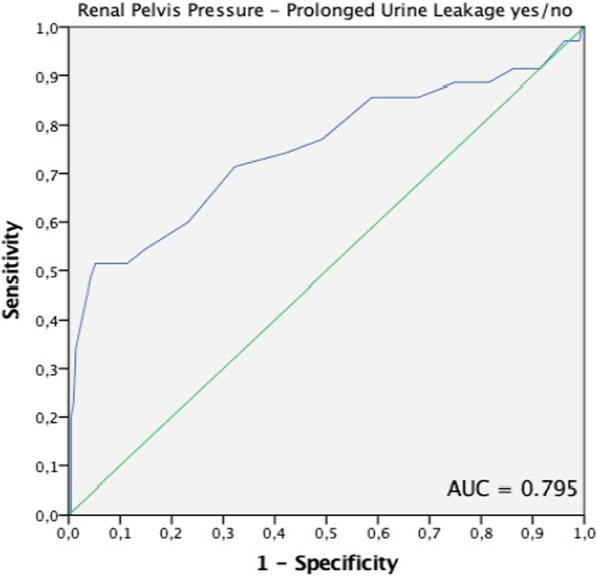
Area under the curve (AUC) for the pressure-controlled nephrostogram. The AUC is 0.795 (95% confidence interval: [0.668; 0.862])

That resulted in a sensitivity of 76.9% (95% CI [60.7%; 88.9%]) and a specificity of 61.5% (95% CI [54.6%; 68.2%]). The negative predictive value (NPV) was 93.4% (95% CI [87.9%; 97.0%]) and the positive predictive value (PPV) 27.3% (95% CI [19.2%; 36.6%]) respectively (Table 2). We evaluated the accuracy of the model by a ROC curve. The area under the curve (AUC) was 0.795 (95% CI [0.668; 0.862]; Fig. 3).

**Table 2:** Overview of the diagnostic accuracy of the PCNG method after PCNL

	Renal pelvis pressure	
	$\le$ 20 cmH_2_O	> 20 cmH_2_O
*Diagnostic accuracy*		
No urine leakage	n = 128	n = 80
Persistent leakage	n = 9	n = 30
Sensitivity [95% CI]	76.9%, [60.7%; 88.9%]	
Specificity [95% CI]	61.5%, [54.6%; 68.2%]	
Negative predictive value [95% CI]	93.4%, [87.9%; 97.0%]	
Positive predictive value [95% CI]	27.3%, [19.2%; 36.6%]	
AUC [95% CI]	0.795 [0.668; 0.862]

While patients in the group with a successful removal of the NT (without PUL) had a significantly lower BMI (27.4 kg/m^2^ vs. 30.7 kg/m^2^, p < 0.001), there was no significantly lower SFR (83.25% vs. 71.79%, p = 0.067).

## Discussion

PUL after removal of the NT after PCNL is a common but minor complication. The most common reason for this event are undetected ureteral fragments causing ureteral obstruction. Ureteral patency can also be impaired because of blood clots, edema of the ureteral wall or the ureteropelvic junction [6]. In this single center retrospective study, we aimed to examine the effectiveness of RPP over FN in order to reduce IR exposure in patients with stones undergoing PCNL.

A short-term leakage can be managed conservatively by repeated dressing changes or by an urostomy bag placed over the wound on the patient’s flank. If this intervention causes any closure of the collecting system, usually an indwelling ureteral stent will be placed for some days in combination with a Foley catheter to promote continuous low-pressure drainage of the bladder.

To detect residual fragments after PCNL in daily practice, postoperative imaging may comprise a CT scan or an antegrade FN. When the contrast medium flows directly into the bladder, an unrestricted patency of the ureter must be assumed. But despite a documented ureteral patency in 100% (n = 248) of the patients in this study, in 16% (n = 39) a PUL was observed. A similar rate (16% of urine leakage > 24 h) is observed by Andonian et al. [6] who assessed the diagnostic utility of FN after PCNL, although they removed NT also in cases of distal ureteral obstruction without evidence of distal stone. Dirim et al. [13] assessed urinary leakage continuously and observed it in 70% of patients from 1 to 200 h. The median duration of leakage was 14 h. In our study, seven patients (3%) required ureteral stent placement after conservative treatment options were unsuccessful, which corresponds to other large studies [14].

Despite good diagnostic accuracy [15] and relevance for clinical decision-making, there is an increasing motivation to reduce patient exposure to IR [7]. Therefore, minimizing additional exposure is particularly important in stone patients because of their unavoidable exposure during the diagnostic and treatment process [8].

The concept of a PCNG was introduced by Robert H. Whitaker, who investigated the dynamics of the upper urinary tract status post renal surgery. The pressure in the kidney was measured at different infusion rates as well as the pressure in the bladder to acquire an “absolute” (renal pelvic) and “differential” (renal pelvis minus bladder) pressure [10]. In a study with 170 pressure flow perfusions Dr. Whitaker discovered that the technique can be useful in patients in a variety of clinical conditions and regardless of their age [9]. Several years later Jeffrey Newhouse established the upper limit for the physiological renal pelvis pressure at 20 cmH_2_O at a perfusion rate of 20 mL/min [12]. His measurements were later confirmed by Veenboer et al. [11].

We were able to demonstrate a significantly lower RPP in patients with an uncomplicated removal of the NT than patients suffering from urine leakage after the removal (p < 0.001). Additionally, in patients who underwent more than one FN due to ureteral obstruction, the RPP after the initial FN was significantly higher than after the second or final FN in the same patient (p < 0.001). Therefore, we show a trend towards lower SFR in patients with PUL and consecutively higher RPP (p = 0.067). Interestingly, patients who had prolonged leakage had a significantly higher BMI (p < 0.001). Despite this finding, we were unable to conclude that higher BMI resulted in a lower stone-free rate (p = 0.234) in an additional analysis, which is in accordance with the literature to date [16].

PCNL is still recommended as the standard of care treatment for large or complex renal stones by current international guidelines [1, 2]. However, to avoid complications of large access (blood loss, pain, need for blood transfusion, etc.), there is a clear trend toward miniPCNL [17]. Endoscopic combined intra renal surgery (ECIRS) is a more recent approach with the goal of minimizing the number of access tracts and improving the one-step SFR [18–20]. ECIRS can be performed in the prone position with the leg divided or in the supine position [21].

Although miniPCNL is associated with a higher rate of tubeless procedures, nephrostomy placement remains the standard of care in larger stones [17]. In ECIRS, the urinary tract (especially in the supine position) can be accessed for residual fragments and is usually stented subsequently with both a Double-J ureteral stent and a nephrostomy tube [18, 21, 22]. In non-tubeless miniPCNL procedures, the RPP should allow the same bedside evaluation of ureteral patency. Limitations arise in its application in ECIRS procedures as long as ureteral stents are placed: since ureteral stents ensure ureteral patency, RPP appears with low clinical relevance.

To avoid FN, there have been multiple studies [23–25] evaluating contrast enhanced ultrasound (CEUS) as a radiation-free alternative to FN in the assessment of ureteral patency after PCNL [23–25]. The results showed that a CEUS nephrostogram can, despite some discordance, assess the drainage into the bladder with statistically comparable results to FN. The authors explained most of the discordance by a higher sensitivity for patency of CEUS compared with FN [23–25]. In contrast to our technique, which can be performed as a bedside test by nursing staff without a long learning curve, both examinations require special equipment and a trained physician, are time-consuming and therefore comparably expensive with US $380 for FN and $320 for CEUS respectively [24].

Another bedside test is the technique of methylene blue (MB) injection combined with capping of the nephrostomy as an alternative to FN. In a prospective trial, the combined technique showed similar accuracy for predicting NT (AUC 0.72 [95% CI 0.61–0.84]) removal after PCNL compared with FN [15]. However, it requires at least 7 mL of MB, which also has relevant costs ($ 50) compared with NSS + i.v.-set ($ 5) used in our technique.

In comparison to the above-mentioned studies, our data show an improved value for the RPP with an AUC of 0.795.

In summary, our data suggest that the measurement of the renal pelvis pressure via the nephrostomy tube provides sufficient accuracy. It is a safe test which is easily performed at the bedside as an alternative to a fluoroscopic nephrostogram. Our study had limitations due to its retrospective design as well lack of control group. In addition, a comparison of both accesses and NT of different sizes would have been interesting.

## Conclusion

The measurement of the RPP appears to provide bedside evaluation of ureteral patency after PCNL. This protocol may help to avoid postoperative radiation exposure and is presumably more cost effective. Future prospective studies will have to validate the clinical application and determine whether provides sufficient accuracy.

## Data Availability

The datasets generated during and/or analyzed during the current study are available from the corresponding author on reasonable request.

## Abbreviations

AUC: Area under the curve
BMI: Body mass index
CVP: Central venous pressure
ECIRS: Endoscopic combined intrarenal surgery
Fr: French
FN: Fluoroscopic nephrostogram
IR: Ionizing radiation
i.v.: Intravenous
NPV: Negative predictive value
NSS: Normal saline
NT: Nephrostomy tube
PCNG: Pressure-controlled nephrostogram
PCNL: Percutaneous nephrolithotomy
POD: Postoperative day
PPV: Positive predictive value
PUL: Prolonged urine leakage
RPP: Renal pelvic pressure
SFR: Stone free rate
